# Monocyte-derived exosomes upon exposure to cigarette smoke condensate alter their characteristics and show protective effect against cytotoxicity and HIV-1 replication

**DOI:** 10.1038/s41598-017-16301-9

**Published:** 2017-11-23

**Authors:** Sanjana Haque, Namita Sinha, Sabina Ranjit, Narasimha M. Midde, Fatah Kashanchi, Santosh Kumar

**Affiliations:** 1 Department of Pharmaceutical Sciences, College of Pharmacy, University of Tennessee Health Science Center, Memphis, TN 38163 USA; 2 Laboratory of Molecular Virology, George Mason University, Manassas, VA 20110 USA

## Abstract

Smoking is known to exacerbate HIV-1 pathogenesis, especially in monocytes, through the oxidative stress pathway. Exosomes are known to alter HIV-1 pathogenesis through inter-cellular communication. However, the role of exosomes in smoking-mediated HIV-1 pathogenesis is unknown. In this study, we investigated the effect of cigarette smoke condensate (CSC) on the characteristics of monocyte-derived exosomes and their influence on HIV-1 replication. Initially, we demonstrated that CSC reduced total protein and antioxidant capacity in exosomes derived from HIV-1-infected and uninfected macrophages. The exosomes from CSC-treated uninfected cells showed a protective effect against cytotoxicity and viral replication in HIV-1-infected macrophages. However, exosomes derived from HIV-1-infected cells lost their protective capacity. The results suggest that the exosomal defense is likely to be more effective during the early phase of HIV-1 infection and diminishes at the latter phase. Furthermore, we showed CSC-mediated upregulation of catalase in exosomes from uninfected cells, with a decrease in the levels of catalase and PRDX6 in exosomes derived from HIV-1-infected cells. These results suggest a potential role of antioxidant enzymes, which are differentially packaged into CSC-exposed HIV-1-infected and uninfected cell-derived exosomes, on HIV-1 replication of recipient cells. Overall, our study suggests a novel role of exosomes in tobacco-mediated HIV-1 pathogenesis.

## Introduction

Approximately 480,000 people in the United States die each year due to the hazards of smoking (Centers for Disease Control and Prevention (CDC), 2017). Cigarette smoke disturbs the redox reaction balance in the body by affecting both antioxidant pathways and reactive oxygen species (ROS) levels. These alterations cause oxidative stress and inflammation, which lead to cellular toxicity and damage in various tissues^1–4^. The oxidative injury results in various pathological complications: respiratory (chronic obstructive pulmonary disease (COPD, asthma), brain (ischemic stroke, Alzheimer’s disease, Parkinson’s disease), cardiovascular systems (coronary heart disease, cardiac stroke), and cancers (lung, cervix, stomach, liver, kidney, and esophagus)^5–13^. A recent study by Mdege. *et al*. (2017) in 28 low-income and middle-income countries has revealed a high prevalence of tobacco use among Human immunodeficiency virus-1 (HIV-1)-infected people^14^. Within the United States, approximately 40% of the HIV-1-infected population are current smokers^15,16^. Despite the use of highly active antiretroviral therapy (HAART), smoking is known to exacerbate morbidity and mortality in HIV-1 patients^16–18^. In HIV-1 patients, smoking further weakens the immune system resulting in a higher risk of virological rebound, an increased rate of immunologic failure, and a decreased response to HAART^19,20^. The progression of smoking-associated diseases is more rapid in HIV-1 infected than in uninfected smokers^21^. Furthermore, several reports also support that smoking enhances HIV-1 infectivity, replication, and its progression to AIDS (acquired immune deficiency syndrome)^22–26^. However, the underlying mechanism of smoking-associated HIV-1 pathogenesis is still under investigation.

Several reports suggest that tobacco exacerbates HIV-1 replication through the oxidative stress pathway^23,24,27,28^. We previously showed that nicotine causes oxidative stress in a cytochrome P450 (CYP)-mediated oxidative stress pathway in HIV-1 model systems; astrocytic and monocytic cell lines^2,29^. We also observed an increased viral load, increased nicotine metabolism, and CYP-mediated oxidative stress in HIV-1 infected smokers compared to non-infected smokers^24,30^. Furthermore, we demonstrated that cigarette smoke condensate (CSC) increases HIV-1 replication in HIV-infected human primary macrophages, perhaps through a CYP-mediated oxidative stress pathway^23,24^. We studied the effect of smoking mainly in monocytes/macrophages because these cells are the secondary targets of HIV-1 and are a major reservoirs for HIV-1^31^. The infected monocytes/macrophages cross the blood-brain barrier (BBB) and infect cells of central nervous system such as perivascular macrophages and microglia^32–34^.

Exosomes are small membrane-bound vesicles with a diameter of <200 nm^35,36^. Exosomes are one of the extracellular vesicles (EVs) that carry various proteins, lipids, mRNA, metabolic enzymes, and miRNAs^37^. They are secreted by most cells into biological fluids and culture media. In past few years, exosomes have gained much attention due to their role in cell-to-cell communication^38–40^. The contents inside exosomes may change under stress conditions such as disease and infection, suggesting their use as therapeutic biomarkers. Exosomes derived from mast cells under stress have extensively different mRNAs, which take part in the protection of recipient cells^41^. Furthermore, exosomes from lymphocytic and monocytic cells are shown to contain miRNA, viral transactivators, and cytokines that affect the course of HIV-1 infection^42–44^. Studies have also shown that exosomes derived from HIV-1 uninfected cells have protective properties, while infected cell-derived exosomes influence infection in uninfected host cells^45,46^. In this study, we examined how exosomes from monocytes communicate with the neighboring HIV-1 infected and uninfected cells to protect smoking-mediated cellular toxicity and viral replication in HIV-1 infected macrophages.

## Results

### Effect of CSC on protein content and antioxidant capacity of U937 cell-derived exosomes

**Figure 1:**
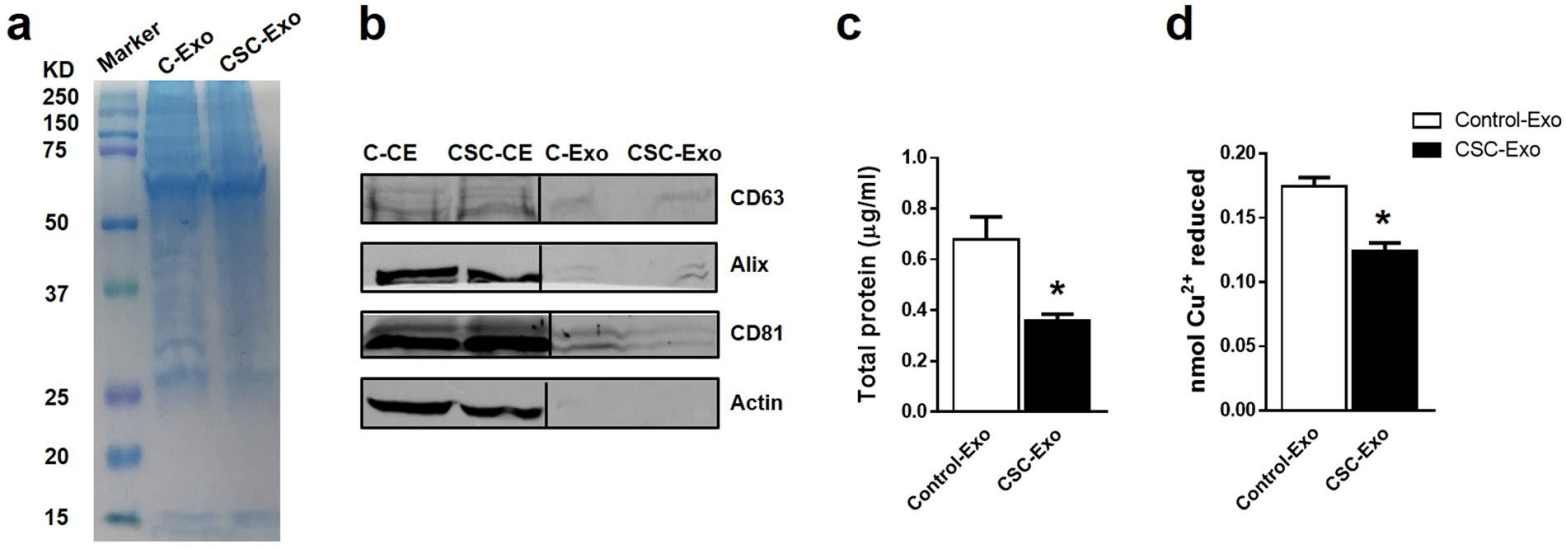
Effect of CSC on U937-cell derived exosomal proteins and antioxidant properties. (**a**) Coomassie blue staining patterns of exosomes that were derived from DMSO as Control or cigarette smoke condensate (CSC)–exposed U937 monocytic cells. C-Exo: Control-exosome, CSC-Exo: CSC-exosome. Equal amount of protein (10 µg) was loaded in each well. (**b**) Western blot of exosomal marker proteins CD63, Alix, and CD81 and cellular marker protein actin. Equal amount of protein (10 µg) was loaded in each lane. C-CE: control-cell extract, CSC-CE: CSC-cell extract, C-Exo: Control-exosome, CSC-Exo: CSC-exosome. (**c**) Total protein levels of exosomes that are quantified using BCA protein estimation method. Bars indicate mean ± SEM values from three replicates. (**d**) Total antioxidant capacity of exosomes measured by total antioxidant capacity assay. *Indicates p < 0.05 compared to control.

Protein extracts from the CSC-treated U937 cells (a model monocytic cells) and exosomes (derived from the CSC-treated U937 cells) were separated on SDS-PAGE gel and stained with Coomassie Brilliant Blue. As shown in Fig. 1a, a relatively less number of proteins and lower amount of individual proteins were observed in exosomes derived from CSC-treated cells (CSC-exosome) than the exosomes derived from the untreated cells (control-exosomes). For the identification of the exosomes, we performed Western blotting using antibodies against CD63, Alix and CD81, which are the protein biomarkers of exosomes (Fig. 1b, for multiple exposure view, refer to supplementary Fig. 1). We observed weak, but detectable bands for all the three proteins in the exosomal pellet, which confirmed the presence of exosomes in the pellet. We did not observe any band for β-actin in exosomal pellets, which further supports our finding. Interestingly, CSC treatment showed an increase in the expression of CD63 and Alix and a decrease in the expression of CD81.

Furthermore, to confirm the altered level of protein in CSC-exosomes (Fig. 1a), the total proteins in the exosomes derived from the treated and untreated cells were quantified. Exosomes from CSC-treated cells showed a significant reduction in the protein level (Fig. 1c, *p < 0.05) compared to control. We also measured whether the altered level of protein in CSC-exosomes changes total antioxidant capacity in the exosomes derived from the treated cells. The results showed that exosomes from CSC-treated cells showed a significant reduction in total antioxidant levels (Fig. 1d, *p < 0.05) compared to control. Thus in the following experiments, we determined whether the decrease in protein level in exosomes by CSC exposure is as a result of a decrease in the total number of exosomes produced by these cells or a decrease in the amount of proteins packaged in each exosome.

### Effect of CSC on physical characteristics of U937 cell-derived exosomes

**Figure 2:**
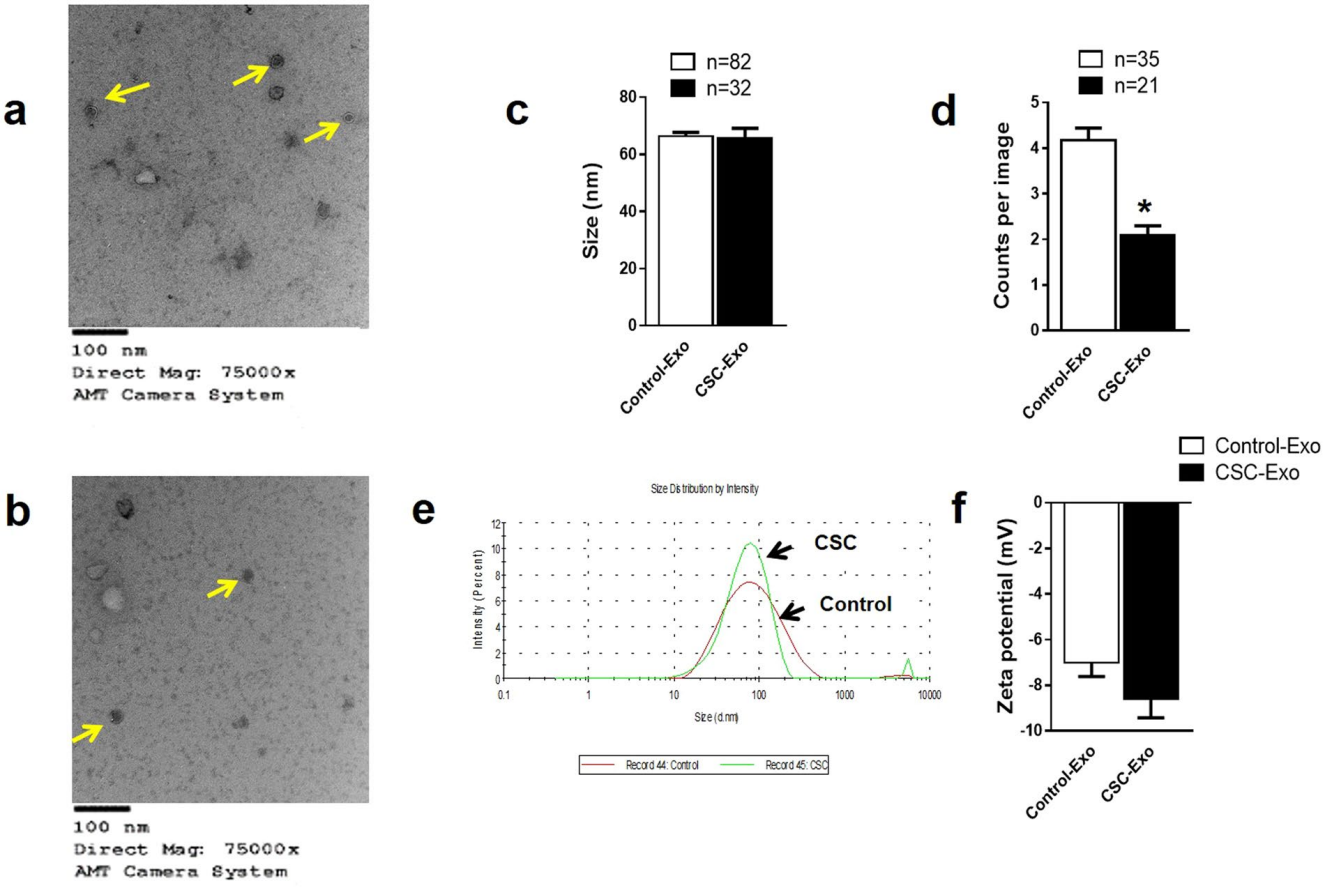
Effect of CSC on physical characteristics of monocyte-derived exosomes. Representative transmission electron microscope (TEM) images of Control (**a**) and CSC-treatment (**b**) derived exosomes. Arrows indicate the vesicles that are considered exosomes with size around 100 nm and intact membranes. Quantitative analysis of exosomal count and size in diameter from three independent experiments are shown in (**c**,**d**), respectively. Bars indicate mean ± SEM values; ‘n’ shows the number of images that were analyzed to measure exosomes count or size. The average hydrodynamic size and size distribution (**e**) and zeta potential (**f**) of exosomes were measured using zetasizer from three independent replicates. *Indicates p < 0.05 compared to control.

Exosomes were characterized by transmission electron microscopy (TEM) and Zetasizer to observe the effect of CSC on the number and size of U937 cell-derived exosomes. Negative staining of samples showed the presence of multiple membrane vesicles with a mean size of 66 ± 1.4 (control) and 66 ± 3.3 (CSC) (Fig. 2a–c). Quantitative analysis from control-exosomes (number of images = 82) and CSC-exosomes (number of images = 32) did not show any significant difference in size (Fig. 2c). However, CSC-exposure (n = 21) significantly reduced the number of exosomes compared to control (n = 35) (Fig. 2d, *p < 0.05). Similar to TEM size distribution, we did not observe any significant variation in mean diameter of the exosomes, when hydrodynamic size was measured using zetasizer (Fig. 2e). However, there is a visual change in the shape of the peaks; exosomes from CSC have a sharper and more concise peak than the control. Additionally, control and CSC-exosomes showed mean negative zeta potentials of −7.0 ± 0.6 and −8.6 ± 0.8, respectively, owing to the negative charge of the phospholipid membrane. Expectedly, CSC treatment did not show any noticeable change to the zeta potential of exosomes compared to the control (Fig. 2f, n = 3). Overall, we observed no significant difference in the size and zeta potential, but a significant decrease in the total number of exosomes upon CSC exposure to U937 cells.

### Effect of CSC on exosomal uptake into the recipient cells

**Figure 3:**
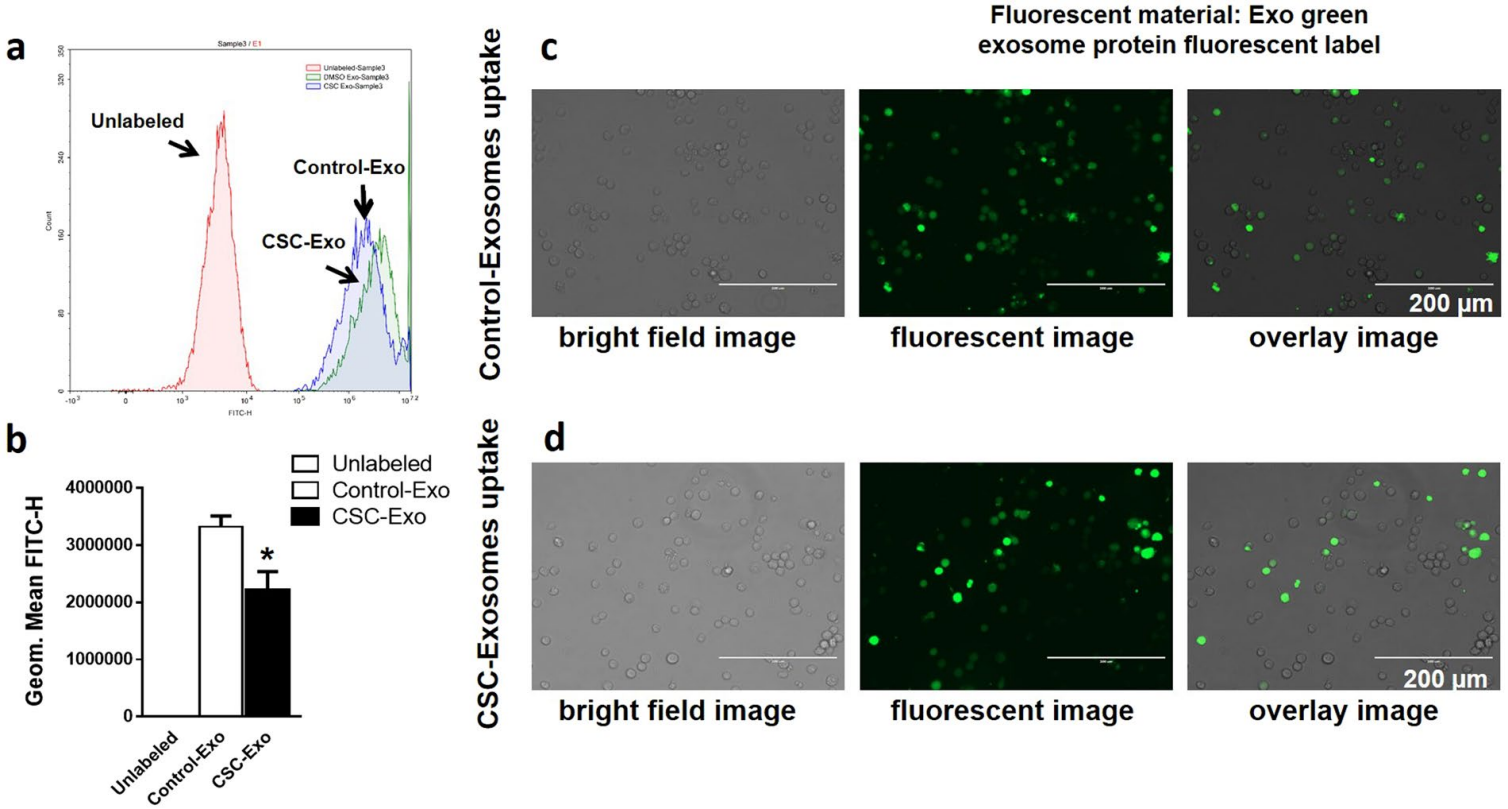
Effect of CSC on exosomal uptake into the recipient monocytic cells. (**a**) Flow cytometry histogram showing the presence of labeled exosomes (10X Exo-Green exosome protein fluorescent label) in the macrophages after 6 hours of exposure. (**b**) Fluorescence intensity in CSC-Exo treated cells compared with DMSO-Exo treated cells. (**c**,**d**) bright field, fluorescent, and overlay images of cells after respective treatments and 6 hours of exposure. Microscope was set to visualize particles of ≤200 µm diameter. Bright field and fluorescent images represent only cells and cells with exosomes respectively. Overlay image combines both of these to demonstrate presence of exosomes within the cells. *Indicates p < 0.05 compared to control-Exo.

For determining exosomal uptake, exosomes isolated from control and CSC-treated plasma were exposed to differentiated U937 cells for 6 hours. The figure obtained from flow cytometry (Fig. 3a) shows a clear difference in the cellular uptakes from the exosomes derived from CSC-exposed cells. From the graphical representation (Fig. 3), it is apparent that the uptake of CSC-exosomes is significantly lower than control-exosomes (Fig. 3b, *p < 0.05). This finding is also supported by the fluorescent microscopy images of the wells (Fig. 3c and d). In the previous section, we demonstrated that CSC exposure causes an overall reduction in the exosomal release (Fig. 2d), which may explain a relatively lower uptake of CSC-exosomes than control-exosomes.

### Effect of CSC/CSC-exosomes on cytotoxicity and DNA damage in U937 cells

**Figure 4:**
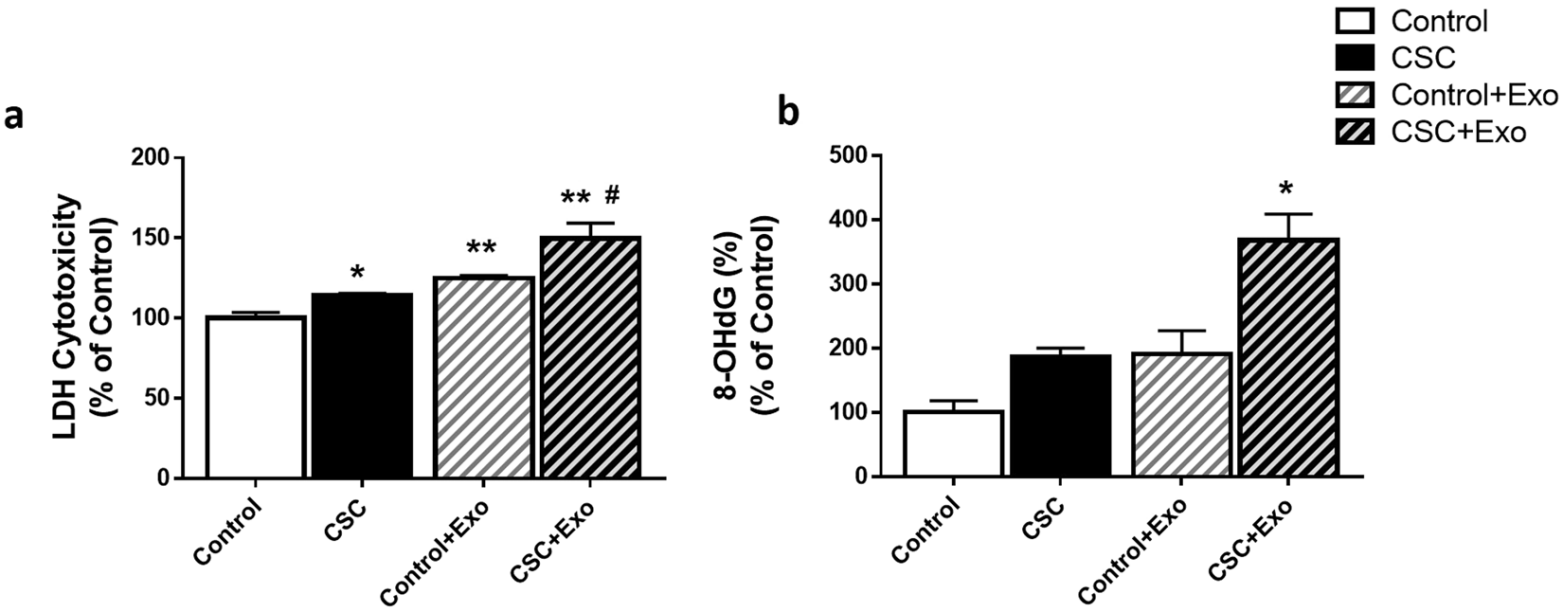
Effect of CSC on cytotoxicity and DNA damage in U937 cells. (**a**) Cytotoxicity level in U937 macrophages after treating them with Control/CSC or exosomes from U937 cell were determined by LDH assay. Macrophages were exposed to U937-exosomes and 10 µg/ml CSC daily dose for 3–4 days daily. Bars indicate mean ± SEM values from three replicates. (**b**) Represents the extent of DNA damage in cells after exposure to Control/CSC or indicated exosomes. We measured 8-OHdG, which is a major product of DNA oxidation. The amount of 8-OHdG detected is directly proportional to the extent of DNA damage due to treatment. All the bars are normalized against the control. Bars indicate mean ± SEM values from two replicates. * and ** indicate p < 0.05 and p < 0.01 compared to control, respectively, ^#^indicates p < 0.05 compared to CSC.

To determine the cytotoxicity caused by CSC/CSC-exosomes on U937 cells, we performed lactate dehydrogenase (LDH) cytotoxicity and DNA damage assay. Differentiated U937 cells were treated with control/CSC and/or exosomes derived from control/CSC-treated U937 cells, respectively. The results obtained from the LDH assay showed that cells treated with CSC-exosomes (exosomes derived from CSC-treated U937 cells) suffered significantly higher toxicity in comparison with the control and CSC-treated cells (Fig. 4a, **p < 0.01 compared to control, ^#^p < 0.05 compared to CSC). CSC and control-exosome treated cells also displayed significantly higher toxicity than the control (*p < 0.05, **p < 0.01). Further analysis showed an additive effect of CSC and control-exosome treatment on the cytotoxicity of U937 cells.

Since CSC and tobacco constituents are known to cause DNA damage^47^, we determined the relative amount of DNA damage by using compound, 8-hydroxy-2′-deoxyguanosine (8-OHdG) in our functional assay. The results showed that CSC-exosome-treated cells had significantly higher DNA damage compared to control as well as CSC-treated and control-exosome-treated cells (Fig. 4b, *p < 0.05). Similar to cytotoxicity, CSC and control-exosome-treated cells also demonstrated a pattern of higher DNA damage than control. Further, the increase in DNA damage by CSC-exosome-treated cells appeared to be higher than the CSC- and control-exosome-treated cells.

### Effect of CSC/CSC-exosomes on exosomal protein content, HIV-1 replication, cytotoxicity, and DNA damage in U1 cells

**Figure 5:**
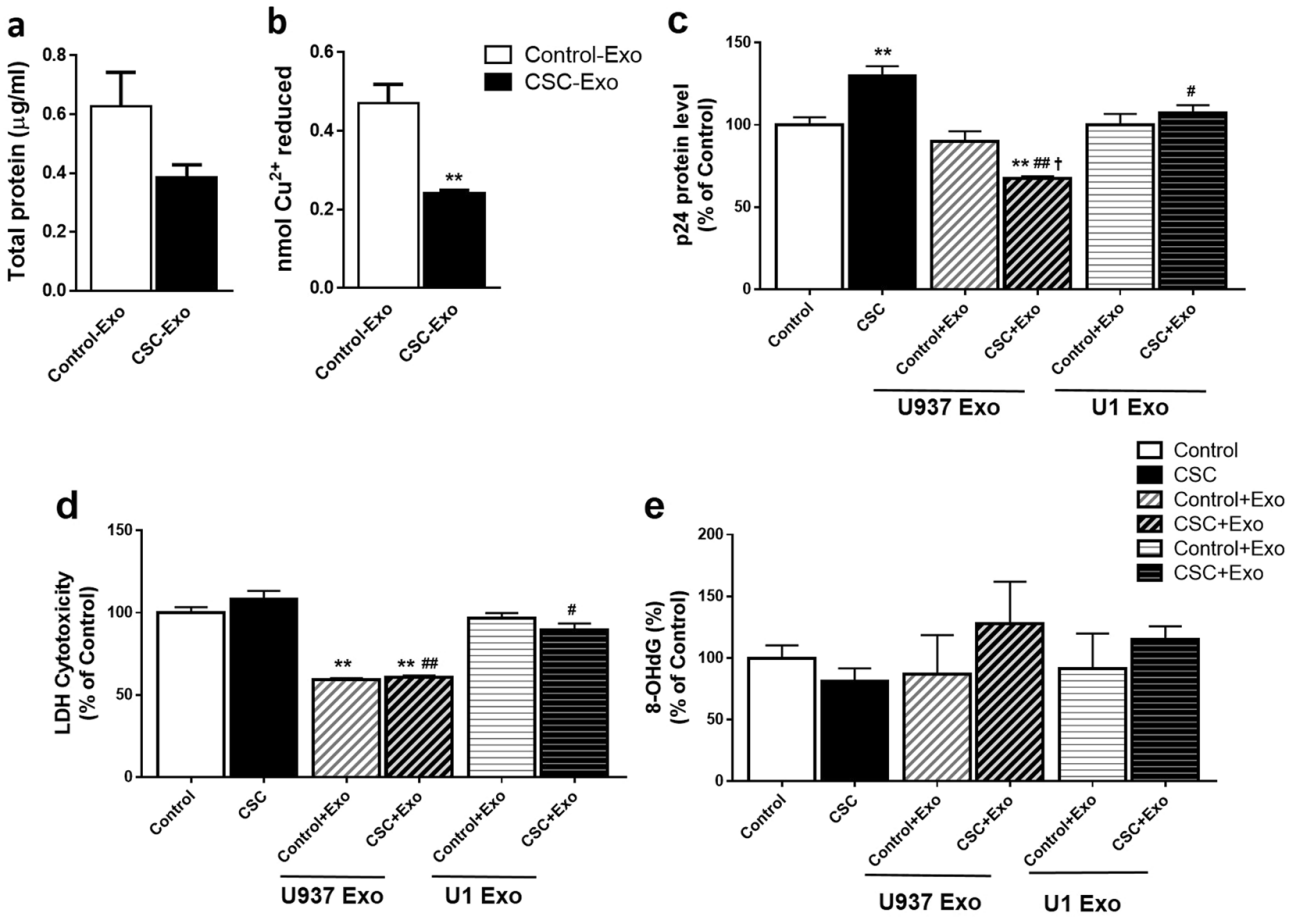
Effect of CSC/CSC-exosomes on exosomal protein content, HIV-1 replication, cytotoxicity, and DNA damage in HIV-1 infected (U1) cells. (**a**) Total protein levels of exosomes from U1 cells treated with control/CSC. Protein quantification was performed by BCA protein assay kit. Bars indicate mean ± SEM values from three replicates. (**b**) Total antioxidant capacity of treated U1 exosomes measured by Total antioxidant capacity colorimetric Assay kit. Bars indicate mean ± SEM values from two and three replicates for control and CSC exosomes, respectively. **Indicate p < 0.01 compared to control. (**c**) HIV-1 p24 levels after treating HIV-1 infected U1 cells with control/CSC or indicated exosomes that were measured by HIV-1 p24 Antigen ELISA kit. (**d**) Cytotoxicity level in U1 cells after exposure to Control/CSC or exosomes from indicated cells were determined by LDH assay. (**e**) DNA damage levels in terms of 8-OHdG% in cells treated with Control/CSC or indicated exosomes. All the bars (Fig. C–E) were normalized against controls. Bars indicate mean ± SEM values from at least three replicates. **Indicates p < 0.01 compared to control, ^#^ and ^##^ indicate p < 0.05 and p < 0.01 compared to CSC, respectively, ^†^indicates p < 0.05 compared to control + Exo.

After observing the impact of CSC/CSC-exosomes on uninfected cells, next we determined the impact of similar treatment on HIV-1 infected U937 cell lines (U1). Prior to CSC/CSC-exosome treatment on U1 cells, we determined whether CSC treatment to the U1 cells alters the total protein and antioxidant levels. Quantification of total protein from exosomes derived from control/CSC-treated U1 cells showed that CSC-exosomes have a lower protein content compared to the control-exosomes (Fig. 5a). As observed in the exosomes obtained from U937 cells (Fig. 1d), CSC-exosomes derived from U1 cells also showed significantly lower antioxidant levels compared to the control (Fig. 5b, **p < 0.01).

HIV-1 replication was measured in U1 macrophages after treating with control/CSC and/or exosomes derived from CSC-treated U937/U1 cells. The p24 level (using ELISA kit) was determined as a measure of viral replication as described earlier^23^. As expected, CSC-treated cells have a significantly higher p24 level than control (**p < 0.01). The exposure of CSC-exosomes from U937/U1 cells to the differentiated U1 cells significantly reduced the viral replication in the later (Fig. 5c, ^#^p < 0.05 and ^##^p < 0.01 compared to CSC). While comparing with CSC treatment, we observed a reduction of 51 ± 3% in p24 level by CSC-exosomes from U937 (CSC-exosome/U937) and a reduction of 29 ± 5% by CSC-exosomes from U1 (CSC-exosome/U1). These results suggest that exosomes derived from uninfected exosomes (exosome/U937) exert more protection from viral replication (p < 0.037). Compared to control, CSC-exosome/U937 treatment reduced p24 significantly (**p < 0.01). In addition, this group showed a significant reduction in the viral load compared to the control-exosome/U937 treated group (^†^p < 0.05).

To investigate the association between the effect of CSC-exosomes on viral load and cytotoxicity, we determined cytotoxicity of CSC-exosome-treated U1 cells. LDH cytotoxicity assay performed on the same treatment groups showed that exosomal exposure to the cells decreased cellular toxicity compared to the control cells (Fig. 5d). While CSC did not show a significant effect on cytotoxicity, the U937 exosome-treated groups showed a significant reduction in cytotoxicity (**p < 0.05) compared to control. When we compared the difference between the CSC treatment group and CSC-exosome/U937 or U1 treatment groups, the CSC-exosome/U937-treated cells showed a significant reduction in cytotoxicity compared to CSC-exosome/U1-treated cells (p < 0.004).

Furthermore, we measured DNA damage in these groups and compared the results with that of cytotoxicity and HIV-1 replication. Though not statistically significant, the U1 cells treated with CSC-exosome/U1 or U937 showed relatively higher DNA damage than the control or only CSC-treated cells (Fig. 5e). On the other hand, the control-exosome/U1 or U937 treated cells showed a pattern of a decreased DNA damage compared to the control treated cells. Overall, we observed that the viral replication and cytotoxicity in U1 cells were relatively more suppressed by exosomes from U937 cells than those from the U1 cells. The results also suggest a positive association between the effects of CSC-exosomes on viral load and cytotoxicity.

### Effect of CSC/CSC-exosomes on HIV-1 replication and cytotoxicity in HIV-1 infected primary human macrophages

**Figure 6:**
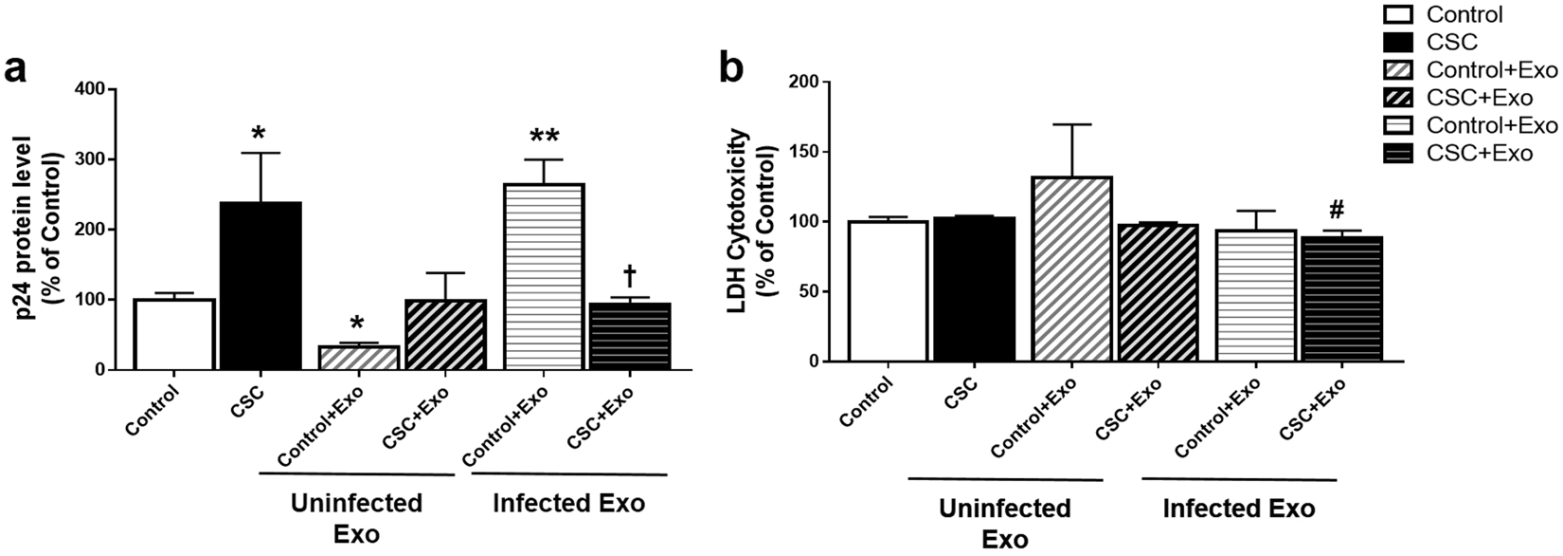
Effect of CSC on HIV-1 replication and cytotoxicity in primary human macrophages. (**a**) HIV-1 p24 antigen level after exposure of CSC/CSC-exosomes to primary human macrophages (PHM). Exosomes were collected from HIV-1 infected/uninfected PHM and introduced to HIV-1 infected PHM along with 10 µg/ml of CSC. (**b**) LDH Assay on primary human macrophages that were treated with control/CSC or indicated exosomes. Bars indicate mean ± SEM values from two replicates. * and ** indicate p < 0.05 and p < 0.01 compared to control respectively, ^#^indicates p < 0.05 compared to CSC. ^†^Indicates p < 0.05 compared to control + Exo within the same group of treatment.

Although U937 and U1 are authentic cell lines for uninfected and HIV-1 infected cell lines, respectively^31,48^, we further investigated the effects of CSC/CSC-exosomes on viral replication and cytotoxicity in HIV-1 infected primary human macrophages (PHM) obtained from a healthy donor. Based on the previously described protocol for exosomal entry into macrophages^49^ and our uptake study on U937-macrophages (Fig. 3), we performed similar study in primary human macrophages. Our results demonstrated an overall protection on viral replication from uninfected CSC-exosomes, which resembles our findings in U1 macrophages. As shown earlier by our group^23,24^, CSC-treated primary macrophages demonstrated significantly higher p24 levels compared to the control (Fig. 6a, *p < 0.05). When the cells were treated with CSC or CSC/Control-exosomes from infected/uninfected primary human macrophages, we observed an overall reduction in p24 levels in the cells treated with CSC-exosomes compared to CSC (Fig. 6a). This finding is similar to the one observed in U1 cells (Fig. 5c). Control-exosomes derived from uninfected primary human macrophages (control-exosome/uninfected macrophages) group showed a significant decrease in viral load compared to the control group. However, in contrast to U1 cells, control-exosomes derived from infected macrophages (control-exosome/infected macrophages) showed a significant increase (*p < 0.05, **p < 0.01). Similar to the U1 cells, CSC-exosome/infected macrophages showed a significant decrease (^†^p < 0.05) in p24, when compared to control-exosome/infected macrophages.

We further investigated a possible association between HIV-1 replication and cytotoxicity upon exposures of CSC/CSC-exosomes to uninfected and HIV-1 infected primary human macrophages. Although not statistically significant, we observed an increase in LDH level in cells treated with control-exosome/uninfected primary macrophages (Fig. 6b). When the cells were treated with CSC-exosome/infected macrophages, the cytotoxicity level dropped significantly compared to the CSC group (^#^p < 0.05). We did not observe much variation in the other treatment groups.

Overall, our results largely validated the effect of CSC, control-exosomes, and CSC-exosomes in HIV-1 infected primary human macrophages. The results suggest that although CSC increases HIV-1 replication, exosomes (derived from both control and CSC-treated cells) from both uninfected and HIV-1 infected cells show a protective effect. Thus, we subsequently studied the exosomal components that may be responsible for the protective effect against cytotoxicity and HIV-1 replication.

### Relative expression of CYPs and AOEs in U937 cell-derived exosomes

**Figure 7:**
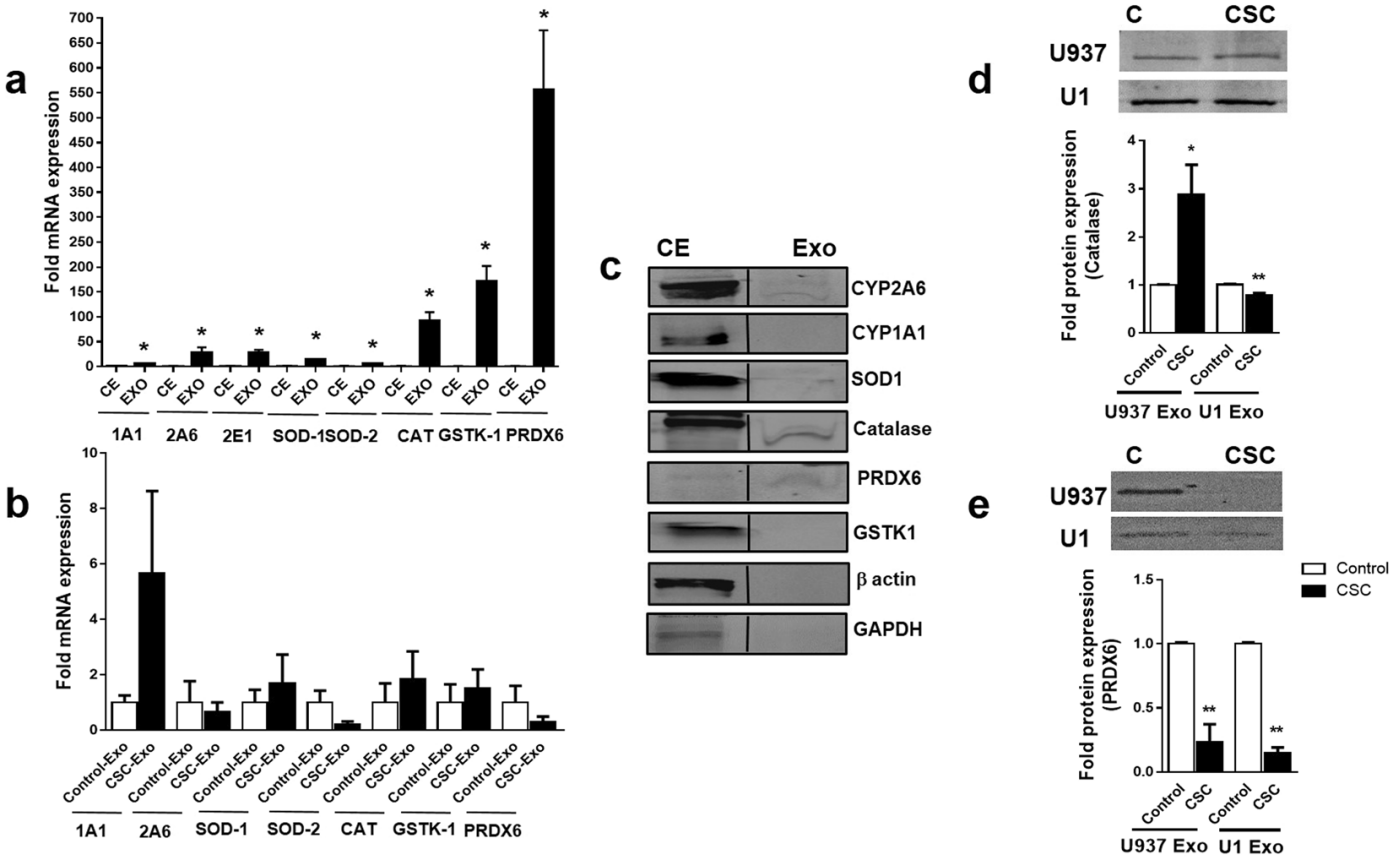
Relative mRNA and protein levels of cytochrome P450s (CYPs) and antioxidant enzymes (AOEs) in monocyte-derived exosomes and effect of CSC on exosomal AOE. (**a**) Relative mRNA levels of CYPs (1A1, 2A6, and 2E1) AOEs (SOD1, SOD2, catalase, GSTK1, and PRDX6) compared with the house-keeping gene GAPDH in exosomes. CE: Cell extract, EXO: Exosome, CAT: Catalase. (**b**) Relative exosomal mRNA levels of CYPs (1A1, 2A6) and AOEs (SOD1, SOD2, catalase, GSTK1, and PRDX6) in exosomes obtained from CSC-treated cells compared to control. (**c**) Representative immunoblots for the expression of exosomal CYPs and AOEs compared to cell extracts. For cell extracts and exosomes, amount of protein loaded was 30 µg and 3 µg respectively. CE: cell extract, Exo: exosome. Effect of CSC on catalase (**d**) and PRDX6 (**e**) levels in exosomes isolated from U937 and U1 cells. Bars indicate mean ± SEM values from three replicates. Equal amount of proteins (4 µg) was loaded on each lane. * and ** indicate p < 0.05 and p < 0.01 compared to each control respectively.

We investigated the potential exosomal components that may provide protection to the monocytic cells. We performed an initial screening of whether U937 monocytic cell-derived exosomes carry CYPs or AOEs (mRNA and/or protein). The role of cellular CYPs and AOEs in tobacco-mediated oxidative stress, cytotoxicity, and carcinogenesis is well-established^48^. Our previous study has also suggested a potential role of CYP and oxidative stress pathways in smoking-mediated HIV-1 replication^24^. We measured the relative level of the mRNAs of CYPs and AOEs in U937 cells and U937-derived exosomes using GAPDH as an internal control for both cells and exosomes. A robust 12- and 27- fold increase in CYP2A6 and CYP2E1 levels, respectively, was observed in exosomes compared to that in cell extract (Fig. 7a, *p < 0.05). Similarly, we observed an increased mRNA expression of AOEs in exosomes than in the cell extract- SOD1 (14-fold), SOD2 (6-fold) (Fig. 7a, *p < 0.05). Exosomes also exhibited 92-, 170-, 555-fold elevation in catalase, GSTK1, and PRDX6 levels respectively (Fig. 7a, *p < 0.05). The results clearly suggest a selective packaging of CYPs and AOEs mRNAs in exosomes compared to the house keeping gene GAPDH. GAPDH is well-accepted house-keeping gene in cells. In exosomes, although GAPDH is not highly expressed, we used this to establish a parameter to compare the selective packaging of the specific genes only. The expression in mRNA level was also altered in CSC-exosomes compared to control-exosomes. The results showed a higher mRNA expression of CYP1A1, SOD1, catalase, and GSTK-1, while a lower mRNA expression of CYP2A6, SOD-2, and PRDX6 in exosomes derived from CSC exposure to U937 cells than in the control (Fig. 7b).

Furthermore, we performed a Western blot for the proteins obtained from the exosomes and their respective cell extract. Although the relative level of protein expression in exosomes compared to cell extract was low, we observed detectable levels of catalase and PRDX6 as well as very low levels of CYP2A6 and SOD1 proteins in exosomes (Fig. 7c, for multiple exposure view, refer to supplementary Fig. 2). Interestingly, the relative level of PRDX6, compared to other proteins, appeared to be higher in exosomes than in the cell extracts even though the same amount of protein was loaded on the gel for the Western analysis of all the proteins/enzymes. As expected, the cellular house-keeping proteins GAPDH and β-actin were negligible/undetectable in exosomes. We then examined whether exosomes derived from CSC-exposed U937 cells alter the level of catalase and PRDX6 in exosomes, and the results are presented in Fig. 7d and e (for multiple exposure view, refer to supplementary Fig. 3). As shown in Fig. 7d, CSC-treatment to U937 cells significantly elevated the level of catalase in exosomes compared to control (*p < 0.05), while slightly decreasing the level of catalase in U1 cells (**p < 0.01). In contrast, a significant reduction of PRDX6 protein was observed in CSC-exosomes (**p < 0.01) both from U937 and U1 cells. These results suggest a potential role of AOEs, especially PRDX6, in protecting the cells from cytotoxicity and HIV-1 replication.

## Discussion

**Figure 8:**
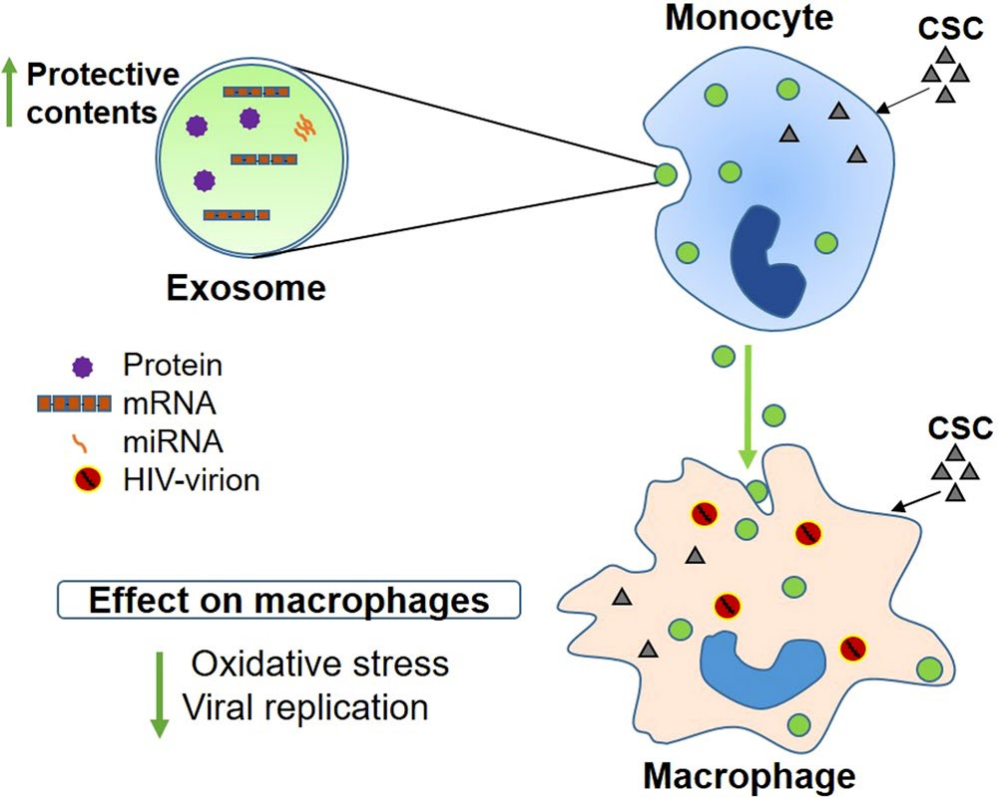
A proposed scheme for delivery of exosomal components to macrophages from monocytes, protecting macrophages from cytotoxicity and viral replication. Exposure to cigarette smoke condensate (CSC) to monocytes causes alteration in the exosomal components such as proteins, mRNA, and miRNA. CSC exposure appears to modify these contents in exosomes, especially antioxidant molecules, and increases the protective capacity of the exosomes. Introduction of the modified exosomes to HIV-1-infected macrophages is proposed to decrease cytotoxicity and HIV-1 replication.

In the present study, we have investigated a protective role of exosomes derived from monocytes against a CSC-mediated increase in cellular toxicity and HIV-1 replication. The findings in this study suggest that exosomes are likely to function as a vehicle to transport protective messages from infected monocytes to the naïve recipient macrophages and protect them from oxidative stress and HIV-1 replication (Fig. 8). This is also an important study on partial physical and biochemical characterizations of exosomes derived from CSC-exposed HIV-1-infected and uninfected monocytes. Initially, we characterized the exosomes derived from uninfected and HIV-1 infected monocytes treated with CSC. These exosomes were further exposed to uninfected and HIV-1 infected macrophages. We observed that exosomes derived from CSC-treated, uninfected macrophages exhibit a better protective capacity against cytotoxicity and HIV-1 replication than those derived from the HIV-1 infected cells.

We used uninfected and HIV-1 infected monocytic cells as well as HIV-1-infected and uninfected primary human macrophages in this study. These cells are model systems for HIV-1 related work. Our recent work with these cells has also shown the effect of CSC and/or nicotine exposure on oxidative stress and HIV-1 replication^23,27^. In this study, we extended our work to establish the role of monocytic exosomes in cellular communication among HIV-1 infected and uninfected cells upon CSC exposure. The characterizations using size determination and zeta potential by zetasizer clearly suggest that the majority of exosomes are within the range of <200 nm. The TEM image, which shows the exosomes in the range of ~100 nm with a transparent double membrane, further validates the quality of these exosomes. The uptake of exosomes by U937-macrophages ensures that exosomes can fuse with the recipient monocytic cells. Finally, the presence of the exosomal marker proteins CD63, Alix, and CD81, together with the absence/negligible level of the cellular marker protein actin, clearly suggests the purity of our exosome samples.

Our analysis of exosomes from CSC-exposed monocytic cells did not show altered size distribution. This result is consistent with earlier studies in which cells treated with cigarette smoke extract, cocaine, or ethanol also did not reveal any effect on exosomal size^23,50,51^. However, we observed a decrease in protein content and antioxidant capacity in the exosomes derived from CSC-treated monocytic cells. Since the average size did not change upon CSC treatment, a decrease in protein content suggests a decrease in the number of exosomes upon CSC exposure.

Cigarette smoke is known to cause oxidative stress, often resulting in DNA damage and ultimately cellular toxicity^23,52^. However, the DNA damage can be repaired depending upon the concentration and time of exposure of the toxicant^53^. In our previous study we observed a significant increase in cellular toxicity after treating monocytes and macrophages with CSC^23^. Similarly, in our current study, we observed an elevation in toxicity and significant loss of cell viability in uninfected macrophages when they were exposed to CSC. Moreover, the cytotoxicity further increased with exposure of exosomes to the macrophages. Exosomes may carry substances that could be toxic to the cells. Lenassi *et al*. have shown that exosomes carry an HIV-1 protein, Nef, which triggers apoptosis in CD4 + T cells^54^. Another study by Emmanouilidou *et al*. also mentions that exosomes are capable of carrying toxic proteins like α-Synuclein, which promotes neurotoxicity in Parkinson’s disease^55^. With CSC and CSC-exosome treatment, we observed an additive increase in cytotoxicity that is associated with an increase in the DNA damage. This result suggests that cytotoxicity through CSC/exosomes could have occurred via DNA damage pathway. DNA damage is known to be a major pathway for tobacco-mediated toxicity^53,56^. In our study, exosomes were observed to carry a lower antioxidant capacity and altered levels of both pro-oxidant and antioxidant enzymes. The mRNA profile of CSC-exosomes from uninfected monocytes indicate that they package a high amount of CYP1A1 which plays a critical role in the metabolism of tobacco constituents. In fact, CYP enzymes have been shown to be associated with oxidative stress that induce cytotoxicity, DNA damage, and viral replication in CSC-treated myeloid cell lines^23^. Additionally, our study demonstrated that PRDX6 level was significantly lower in CSC-exosomes, however, the catalase level was observed to be high. The role of PRDX6 as an antioxidant is known to suppress lipid oxidation and therefore PRDX6 helps in rescuing cellular toxicity from external agents^57–59^. Perhaps, it is possible that increased CYP1A1 expression and decreased PRDX6 level in exosomes could increase oxidative stress and resultant DNA damage and cellular toxicity.

Interestingly, exosomes derived from the CSC-treated uninfected monocytic cells conferred protection against HIV-1 replication in U1 cells. It has been observed in previous studies that exosomes derived from cells undergoing stress can be protective to recipient cells from further cellular damage^60^. This is the first study to show the similar protective effect by exosomes in HIV-1-infected cells. However, the viral suppression is diminished when the cells were treated with CSC-exosomes from HIV-1 infected cells. It could be possible that these exosomes provide protection against viral replication during the early stage of HIV-1 infection. However, with the progression of the disease, the protective capacity of the exosomes may be compromised. The decreased cytotoxicity level in the same cells, after the treatment of CSC-exosomes from uninfected monocytes, and further increase in cytotoxicity after treatment of CSC-exosomes from infected cells, also corroborates our hypothesis. The reason for higher protection against cytotoxicity provided by U937 cell-derived CSC-exosomes compared to U1 cell-derived CSC-exosomes can be explained by their catalase content. U937 cell-derived CSC-exosomes package higher catalase protein, suggesting higher protection to recipient cells. On the contrary, U1 cell-derived exosomes inherently contains lower catalase and PRDX6 protein, resulting in compromised protection against cellular toxicity and subsequently viral replication. These observations further provide an association between cytotoxicity and viral replication, which is likely to be mediated through altered levels of antioxidant enzymes.

Although monocytes and macrophages are well-accepted cell lines for studying HIV-1 disease, the effects observed with these cell lines may not translate fully into primary macrophages. To validate our finding from cell lines, we utilized uninfected and HIV-1 infected primary human macrophages. As these cells are directly derived from healthy patients’ blood, they are less vulnerable to stimulated oxidative stress and viral replication compared to U937 and U1 cells. Interestingly, CSC-exosomes from HIV-1 infected cells also provided protection against viral replication in infected cells. This protection may have resulted from a difference in the formation of exosomes upon CSC exposure to acutely-infected primary macrophages vs. constitutively-infected U1 cells.

CYP enzymes have been known to be involved in the metabolism of numerous xenobiotics, including the metabolism and activation of polycyclic aromatic hydrocarbons, largely found in cigarette smoke^1,61^. In our previous studies, we observed elevated ROS production and resulting oxidative stress followed by apoptotic cell death, in monocytic cells after treatment with CSC^23^. CYP enzymes are sufficiently expressed in these cells, but the underlying mechanistic pathway through which exosomal CYPs may affect the neighboring cells is unknown. The current study revealed that exosomes from naïve cells express relatively high levels of CYP1A1, CYP2A6, and CYP2E1 compared with the house-keeping genes GAPDH or actin. This may explain the increased cytotoxicity that we observed after introducing CSC-exosomes in U937 cells. In addition to the CYPs, the exosomes also expressed higher levels of AOEs such as SOD1, SOD2, catalase, GSTK1, and PRDX6 compared to the house-keeping gene. We have also performed proteomics on U937-macrophage-derived exosomes and observed the presence of these proteins, which further validates our Western data. There was an increase in the expression of catalase in exosomes derived from CSC-exposed U937 cells, however the expression of PRDX6 was lowered. The modulation of these antioxidants in the exosomes upon CSC exposure could have an impact on overall oxidative stress and subsequent cytotoxicity and viral replication in the recipient cells. However, other exosomal contents such as miRNAs and inflammatory cytokines are also possible factors for triggering oxidative stress in the recipient cells^62,63^. We will further characterize the exosomal contents using proteomics, nanostring technology, and cytokine profiling to investigate their role in oxidative stress and HIV-1 replication.

Nicotine in cigarette smoke has been shown to suppress inflammation in various cells by expressing pro-inflammatory cytokines such as TNF-α, IL-6, IL-12 and IL-1β though nicotinic acetylcholine receptors (nAchRs)^64–67^. Although nAchRs are known to be present in monocytic cells^68^, our study did not show detectable levels of nAchRs in exosomes derived from HIV-1 infected or uninfected monocytic cells. Thus, we present exosomes as novel protective agents that likely transport CYPs and AOEs from the naïve monocytic cells and protect the HIV-1 infected cells from CSC-mediated oxidative stress and viral replication. In addition to its anti-inflammatory role, nicotine at a low dose is also reported to upregulate the expression of AOEs to combat the cellular oxidative stress^69^. Although exosomes derived from nicotine-exposed cells have not been characterized, it is possible that the exosomes contain nicotine-induced AOEs that may be responsible for protecting the recipient cells. A recent study that has shown that exosomes are capable of transporting the AOEs from one cell to other to render protection to the recipient cell, further corroborates the findings from our study^70^.

In conclusion, this study demonstrates that exosomes secreted from monocytes upon exposure to CSC alter the exosomal protein level, antioxidant capacity, and packaging of antioxidant enzymes. The study also suggests that exosomes derived from CSC-exposed monocytic cells, especially uninfected monocytes, have protective effects against toxicity and viral replication (Fig. 8). The findings from this study have clinical relevance with respect to utilization of these nano-vesicles as therapeutic carriers to suppress oxidative stress-induced cytotoxicity and HIV-1 replication. Further studies to characterize specific proteins, miRNA, or metabolites in exosomes may provide specific biomarkers for CSC-mediated effects on cytotoxicity and HIV-1 pathogenesis. These components may also be packaged inside the exosomes, and are delivered to naïve cells for specific biological effect.

## Methods

### Cell culture and treatment

Two monocytic cell lines- U937 cells from ATCC (Manassas, VA) and the HIV-1 infected U1 cells from the NIH AIDS Reagent Program (Germantown, MD) were used in this study. Cells were treated with 10% exosome-depleted FBS (Exo-FBS) containing Roswell Park Memorial Institute (RPMI) 1640 media^71^. The media was supplemented with 2% sodium bicarbonate, 1% L-glutamine, and 0.1% gentamicin^1^. Cells (0.1 million/ml) were incubated in 6-well plates at 37 °C with 5% CO_2_, and then treated everyday with CSC (10 µg/ml) and Dimethyl sulfoxide (DMSO) as control for 4 days. Our previous studies have shown that acute treatment of 50 µg/ml CSC, which is near the physiological concentration of nicotine and other constituents, increases the ROS level, without appreciable toxicity^23,72^. For the current study, we chose a CSC concentration that would accumulate to 40 µg/ml after 4 days of treatment, and would generate minimal toxicity and sufficient exosomes for further analysis. Exosomes were isolated from 1 ml supernatant from each well and were exposed to differentiated U937 and U1 cells with 1 ml media. Our exosomal treatment was based on volume/volume rather than protein amount to closely reflect physiological condition. For differentiation, U937 and U1 cells (0.8 million/ml) were seeded in 6-well plates with RPMI media including 80 nM phorbol 12-myristate 13-acetate (PMA). After 3 days, media and non-adherent cells were removed by washing with phosphate buffer saline (PBS) followed by overnight incubation with fresh RPMI media containing Exo-FBS^23^. The differentiated U937 and U1 cells were treated with DMSO or CSC, as well as with the exosomes that were isolated from the same amount of media from each control and CSC-treated cells for 3–4 days.

### Collection of peripheral blood mononuclear cells (PBMC) and HIV-1 infection

PBMCs were isolated using density gradient fractionation from the buffy coat from de-identified healthy subjects who are free from infections, namely HIV-1, and are free from drugs abuse, especially tobacco smoking (Interstate Blood Bank Inc., Memphis, TN)^23^. All the procedures were performed under the guidelines of International Review board (IRB). The monocytes were separated from whole blood by RosetteSep Human Monocyte Enrichment Cocktail^73^ layered on ficoll and centrifuged at 1200 g for 20 minutes. The white ring of PBMCs were collected carefully, washed a few times to remove ficoll, and incubated with Ammonium-Chloride-Potassium buffer to lyse red blood cells. Following an overnight incubation with RPMI media containing human serum, the media was replaced with fresh media supplemented with macrophage colony stimulating factor (50 ng/ml) to promote differentiation into macrophages^74^. After 7–10 days, macrophages were collected and treated with polybrene (2 µg/ml) and IL-2 (interleukin-2, 10 ng/µl), and then infected with HIV-Ada strain at 20 ng/10^6^. Infected and uninfected cells were seeded in 6-well plates and fresh media was added every 3–4 days. The infection level (p24 antigen) was measured using an ELISA kit^23^. Once the infection was confirmed, the cells were cultured in fresh RPMI media containing Exo-FBS and treated with Control/CSC and/or exosomes as described above.

### Preparation and isolation of exosomes

Supernatant from cell culture media was used to isolate exosomes by Invitrogen-Total Exosome Isolation (from cell culture media) kit (Life Technologies, NY) and using ultracentrifugation as described previously^75,76^. Briefly, supernatant was centrifuged (2000g for 30 minutes) and Total exosome isolation reagent was added to the supernatant followed by overnight incubation at 2°–8 °C. After an hour of centrifugation (10,000 g, 2°–8 °C) exosomal pellet was obtained. For ultracentrifugation, initially the cells were centrifuged at lower speeds (300 g and 2000g for 10 minutes each) followed by higher speeds (10,000 g for 30 minutes and 100,000 g for 70 minutes) to remove cell debris. The pellets containing exosomes were washed with PBS and centrifuged again (100,000 g for 70 minutes). The ultracentrifugation step is tedious and requires >10-times media; therefore, it was avoided for experiments that needed large amount of exosomes. To determine the impurities of HIV-1/viral proteins, we measured the relative amount of a viral p24 protein in the U1-cell derived exosomes and compared it with the U1 media. Exosomes carry 10–15 times less amount of p24 compared to the media. The presence of detectable p24 in the exosomes may be as a result of a 5–10% impurity in isolation method or packaging of p24 in exosomes. This finding suggests that p24 present in exosomes is unlikely to affect our results. This is also reflected in our finding, in which, the exposure of exosomes from U1/HIV-infected primary macrophages to naïve U1/HIV-infected primary macrophages showed decrease in the viral load (Figs 5c and 6a). These exosomes were further characterized using zetasizer, transmission electron microscopy (TEM), and exosomal marker proteins (CD63, CD81 and Alix).

### Electron microscopy and Dynamic light scattering

Exosomes were analyzed by Zetasizer Nano-ZS (Malvern Instruments Inc, Malvern, UK) as described previously^77^. The exosomes were dissolved in 1 ml ultrapure water and 1 ml PBS for determining size and zeta potential, respectively. To further confirm the size, shape, and quality of exosomes from cultured media, we employed JEOL 2000EXII TEM (The Neuroscience Institute, University of Tennessee Health Science Center). Freshly prepared exosomes were resuspended in 1X PBS and loaded on slides as described^78^.

### Exosomes labeling, uptake, and flow cytometry

The uptake of exosomes by U937 macrophages was monitored by using Exo-GLOW^TM^ Exosome Labeling Kits (System Biosciences, CA). Briefly, exosomal pellet containing 100–500 µg protein was suspended in 500 µl 1X PBS, with subsequent addition of 50 µl 10X Exo-Green exosome protein fluorescent label. The exosome solution was incubated at 37 °C for 30 minutes followed by the addition of 100 µl ExoQuick-TC to stop the labeling reaction. The labeled pellet was exposed to U937 macrophages and their uptake by the cells at different time points was visualized under fluorescent microscope and flow cytometry.

### DNA, RNA, and protein isolation

DNA and RNA were isolated from cells using DNeasy Blood & Tissue Kit (50) and RNeasy Mini kit (250) (QIAGEN, Germantown, MD) respectively^1^. Exosome RNA and Protein isolation Kits (Life Technologies, NY) were used to obtain RNA and protein from the exosomes. Nanodrop 2000c Spectrophotometer (ThermoFisher Scientific, Rockford, IL) was utilized for quantifying RNA and DNA. All the procedures were performed following the suppliers’ protocols.

### Quantification of protein and antioxidant capacity

Protein from cells and exosomes was quantified by BCA protein assay kit (ThermoFisher Scientific, Rockford, IL). The protein isolated from exosomes was subjected to Antioxidant Capacity Assay, using Total Antioxidant Capacity colorimetric Assay Kit (BioVision, Milpitas, CA) as described by the manufacturer.

### Cytotoxicity

For measuring the cytotoxicity caused by the respective treatment, we used the Pierce^TM^ LDH Cytotoxicity Assay Kit (Thermo Scientific, Rockford, IL) as described by the manufacturer. This assay measures extracellular lactate dehydrogenase (LDH) in the media, which is indicative of cellular damage. The supernatant was incubated with LDH reaction mixture and the optical density was measured at 490 and 680 nm.

### DNA Damage

The compound, 8-hydroxy-2′-deoxyguanosine (8-OHdG) is a common marker for DNA damage^79^. EpiQuik^TM^ 8-OHdG DNA Damage Quantification Direct Kit (Colorimetric) (EPIGENTEK, Farmingdale, NY) was used to measure the 8-OHdG level following the manufacturer’s protocol. Briefly, DNA was bound to the wells by binding solution and incubated sequentially with capture and detector antibody. After addition of the developer solution, the signal was measured at 450 nm^24^.

### Viral Load Count

The viral load was determined in the supernatant collected from U1 and primary macrophages (post treatment with control/CSC and/or exosomes) by using the HIV-1 p24 Antigen ELISA kit (Zeptometrix Corporation, Buffalo, NY)^23^.This assay measures the level of p24-a structural protein of HIV-1. Briefly, the viral antigen was captured on the immobilized antibody, followed by a reaction with biotin conjugated human anti-HIV-1 antibody and subsequent incubation with Streptavidin-Peroxidase. The reaction between the substrate and antibody was measured at 450 nm. We used a standard curve for p24 to determine the viral load (p24) from our samples.

### Quantitative reverse transcriptase polymerase chain reaction (RTPCR)

Quantitative RTPCR was employed to determine the relative mRNA fold expression level following the protocol described in our previous studies^1,23^. Briefly, purified RNA from cell extract (80–120 ng) and exosomes (10–20 ng) was reverse transcribed to cDNA by SimpliAmp Thermal Cycler (Applied Biosystems, Foster City, CA) and was amplified in a Step-One Plus Real-Time PCR System (Applied Biosystems, Foster City, CA) using TaqMan Gene Expression kit (Applied Biosystems, Foster City, CA). We determined the mRNA expression level of CYPs 1A1 (Hs01054794_m1), 2A6 (Hs00868409_m1), 2E1 (Hs00559368_m1) and the antioxidant enzymes (AOEs) superoxide dismutase (SOD1 (Hs_00533490m1) and SOD2 (Hs00167309_m1)), catalase (Hs00156308_m1) and Glutathione S-transferase kappa 1 (GSTK1) (Hs00210861_m1). We tested the expression of a known exosomal marker RNA (U6snRNA) and cellular house-keeping genes, actin and GAPDH, to analyze the relative expression of exosomal mRNAs compared to cells, and the effect of CSC exposure on the expression these mRNAs. The cycle numbers of U6snRNA and actin were undetermined and very low, respectively. However, the expression of GAPDH mRNA was low but sufficient (<40 cycles) that could be measured without much differences between the samples. Therefore, we used GAPDH as an internal control for both cellular and exosomal mRNAs. As expected, the relative level of GAPDH mRNA expression in exosomes is much lower than that of cellular GAPDH mRNA. The relative expression of cellular and exosomal mRNAs were calculated using the fold expression for these genes by 2^−ΔΔCt^ method.

### Western Blotting

To determine the expression of protein, approximately 20–25 µg of protein from cell extracts and 5–10 µg protein from exosomes were loaded into polyacrylamide gel (4% stacking, 10% resolving gel). Upon running the gel, the proteins were transferred to a polyvinyl fluoride membrane and were blocked with 5–10 ml of Li-Cor blocking buffer (LI-COR Biosciences, Lincoln, NE) for 1 hour. The membranes were incubated overnight with primary antibodies (AHR Mouse Mab, ThermoFisher, Rockford, IL; GAPDH, CYP1A1, CYP2A6 Rabbit Mab, Abcam, Cambridge, MA; SOD1, Catalase, CD63 Mouse Mab, CD81 Rabbit Mab, GSTK1 Goat Mab, Santa Cruz, Dallas, TX; PRDX6 Rabbit Mab, LS Bio Seattle, WA; β actin Rabbit Mab, Cell Signaling, Danvers, MA; Alix Rabbit Mab, Protein Tech, Rosemont, IL) at 4 °C. The antibody dilution ranged from 100 to 500 times as appropriate. The next day, the membranes were washed and incubated with respective secondary antibodies (Goat anti-Mouse Mab, Goat anti-Rabbit Mab, Donkey anti-Goat Mab) and the signal was detected using LI-COR (Biosciences, Lincoln, NE). To determine the fold expression of the proteins, the densitometry data was obtained from Image Studio Lite version 4.0.

### Statistical analysis

For the data analysis, mean ± SEM was calculated and normalized against the control group. Two-tailed t-tests were used to calculate the statistical significance. All the statistical calculations were performed using GraphPad Prism 7 (San Diego, CA).

## Electronic supplementary material

Supplementary Figures
