# Supplementary material for: Monocyte-derived exosomes upon exposure to cigarette smoke condensate alter their characteristics and show protective effect against cytotoxicity and HIV-1 replication
Source: Sci Rep. 2017 Nov 23;7:16120. doi: 10.1038/s41598-017-16301-9 (PMC5701054; doi:10.1038/s41598-017-16301-9)
Supplement: Supplementary file 1 — Supplementary Figures [file 41598_2017_16301_MOESM1_ESM.pdf]

Supplementary figures

Monocyte-derived exosomes upon exposure to cigarette smoke condensate alter their characteristics and show protective effect against cytotoxicity and HIV-1 replication

Sanjana Haque[1], Namita Sinha[1], Sabina Ranjit[1], Narasimha M. Midde[1], Fatah Kashanchi[2], and Santosh Kumar[1*]

[1]Department of Pharmaceutical Sciences, College of Pharmacy, University of Tennessee Health Science Center, Memphis, TN 38163

[2]Laboratory of Molecular Virology, George Mason University, Manassas, VA 20110

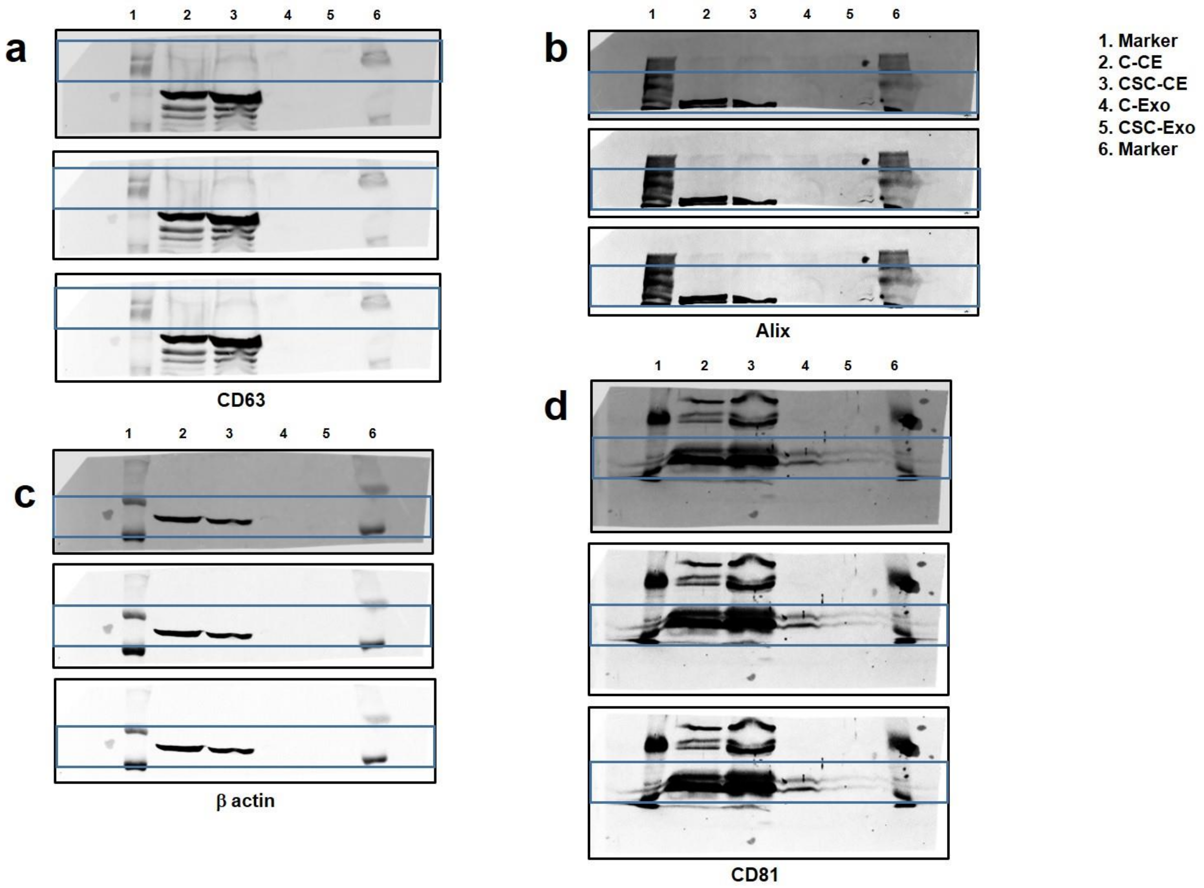

Supplementary figure 1: Western blot of exosomal and cellular marker proteins. (a) CD63 **(b)** Alix **(c)** β actin **(d)** CD81. Each blot is presented in original and multiple contrast exposure sequentially from top to bottom.

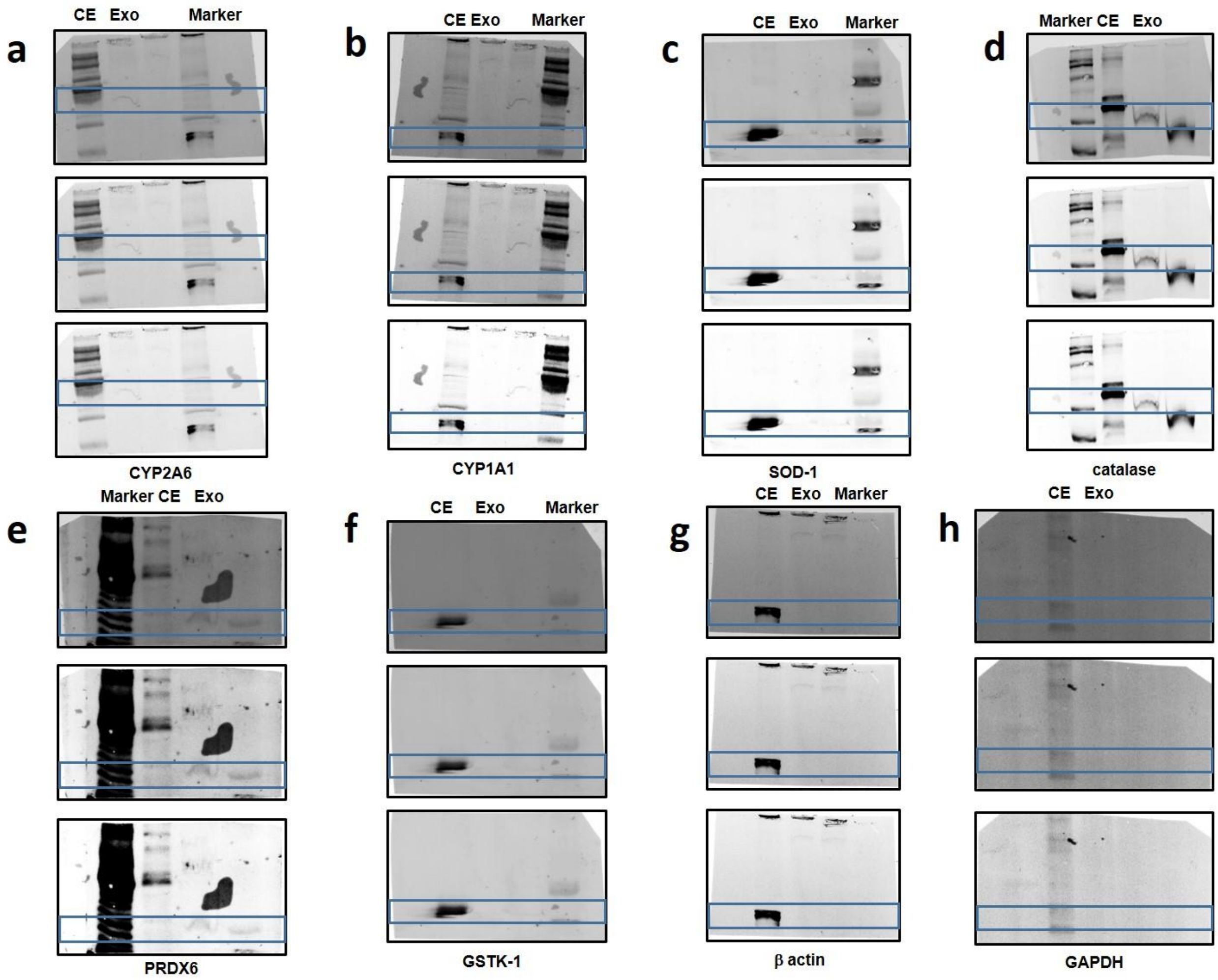

Supplementary figure 2: Representative immunoblots for the expression of exosomal CYPs and AOEs compared to cell extracts. (a) CYP2A6 **(b)** CYP1A1 **(c)** SOD-1 **(d)** catalase **(e)** PRDX6 **(f)** GSTK-1 **(g)** β actin **(h)** GAPDH. Each blot is presented in original and multiple contrast exposure sequentially from top to bottom. CE: cell extract, Exo: exosomes

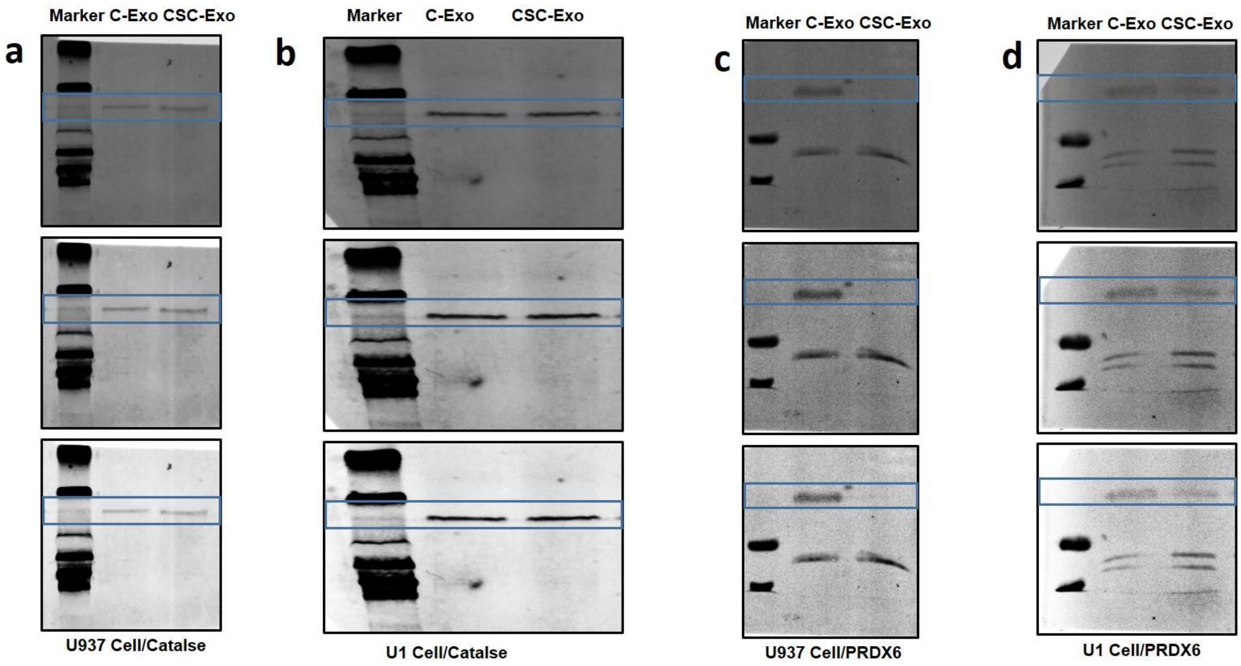

Supplementary figure 3: Effect of CSC on specific AOE levels in exosomes isolated from U1 and U937 cells. (a) catalase from U937 cell extract and exosomes **(b)** catalase from U1 cell extract and exosomes **(c)** PRDX6 from U937 cell extract and exosomes **(d)** PRDX6 from U1 cell extract and exosomes. Each blot is presented in original and multiple contrast exposures sequentially from top to bottom. CE: cell extract, Exo: exosomes.
